# Is There Evidence of Crohn’s Disease Exclusion Diet (CDED) in Remission of Active Disease in Children and Adults? A Systematic Review

**DOI:** 10.3390/nu16070987

**Published:** 2024-03-28

**Authors:** Inês Correia, Patrícia Almeida Oliveira, Maria Luz Antunes, Maria da Graça Raimundo, Ana Catarina Moreira

**Affiliations:** 1 ESTeSL—Escola Superior de Tecnologia da Saúde de Lisboa, Instituto Politécnico de Lisboa, 1990-096 Lisboa, Portugal; paaoliveira@ulslo.min-saude.pt (P.A.O.); mluz.antunes@estesl.ipl.pt (M.L.A.); mraimundo@hevora.min-saude.pt (M.d.G.R.); ana.moreira@estesl.ipl.pt (A.C.M.); 2 Faculdade de Medicina, Universidade de Lisboa, 1649-028 Lisboa, Portugal; 3 Hospital do Espírito Santo de Évora, EPE, 7000-811 Évora, Portugal; 4 H&TRC—Health & Technology Research Center, ESTeSL—Escola Superior de Tecnologia da Saúde, Instituto Politécnico de Lisboa, 1990-096 Lisboa, Portugal; 5 APPsyCI—Applied Psychology Research Center Capabilities & Inclusion, ISPA—Instituto Universitário, 1149-041 Lisboa, Portugal

**Keywords:** Crohn’s disease, exclusive enteral nutrition, partial enteral nutrition, Crohn’s disease exclusion diet, remission

## Abstract

Crohn’s disease (CD) is an inflammatory bowel disease. Previous research has explored the impact of diet on CD, as specific dietary components can influence gut microbiota and immune responses, contributing to damage in the gastrointestinal tract. The Crohn’s Disease Exclusion Diet (CDED) is based on an exclusion diet; it is a recent dietary approach that is often used alongside partial enteral nutrition (PEN) and aims to induce disease remission by excluding certain dietary components. This study assesses the current evidence for the effectiveness of the CDED + PEN in achieving remission in both children and adults with active CD. Our systematic review followed PRISMA recommendations and was registered in PROSPERO with CRD number 42022335076. The searched databases were PubMed/MEDLINE, Cochrane Library, Scopus, and Web of Science. The included studies were analyzed using Rayyan software, and the risk of bias was assessed with Cochrane RevMan 5.0 software. The primary assessed outcome was clinical remission, evaluated with validated questionnaire scores such as PCDAI, CDAI, or HBI. All analyzed papers yielded promising results. Notably, the CDED + PEN demonstrated better tolerance than exclusive enteral nutrition (EEN), resulting in higher adherence rates. Therefore, the CDED + PEN appears to be a viable alternative for induction remission in active disease for both children and adults with CD.

## 1. Introduction

Crohn’s disease (CD) is a chronic disease, part of inflammatory bowel disease, which primarily affects the intestine, particularly the ileum and colon. It results from an amplified immune response to intestine’s contents in genetically susceptible individuals [1,2,3,4,5].

It can be categorized based on age at diagnosis, location, and behavior of the disease. In children, classification also considers its impact on growth and development. Disease activity is classified as mild, moderate, or severe, using indexes like the Crohn’s Disease Activity Index (CDAI) or the Harvey–Bradshaw Index (HBI) for adults, and the Pediatric Crohn’s Disease Activity Index (PCDAI) for children and young people [6,7,8,9,10,11].

CD is a global disease with increasing worldwide incidence and prevalence [12,13,14]. Its causes are a complex interplay of genetic and environmental factors, leading to immune system dysregulation and gastrointestinal damage [1,5,15,16].

The environment directly influences the intestinal microbiome. For instance, modern lifestyles characterized by diets high in lipids, sugars, gluten, dairy products, food additives, and low fiber can compromise the intestinal barrier, causing dysbiosis and increased permeability [15,17,18].

In children with mild-to-moderate CD, the first-line treatment recommended by the European Crohn’s and Colitis Organization (ECCO) and the European Society for Paediatric Gastroenterology Hepatology and Nutrition (ESPGHAN) is exclusive enteral nutrition (EEN) [19]. For adults, treatment typically includes corticosteroids, immunomodulators, and/or biological therapies [20].

In pediatric patients, EEN may not significantly differ from corticosteroid treatment in terms of achieving clinical remission, but it consistently demonstrates superior efficacy in promoting mucosal healing and positively impacting the growth and development of the child [19,21,22,23,24,25].

Although the precise mechanism by which EEN induces remission in CD remains uncertain, it is understood that EEN directly restores the function of the epithelial barrier, reduces permeability, prevents bacterial invasion into the mucosa, and reduces inflammation by lowering pro-inflammatory cytokines [15,25,26].

Studies suggest that polymeric formulas are the most commonly used enteral formulas, probably due to their better taste and lower cost [25,27,28].

However, despite the advantages of EEN, there are limitations to consider. EEN is intended for short-term use, lacks a remission maintenance strategy, and requires complete avoidance of all solid foods, which can pose challenges for adherence [19,24,25,29,30].

Since EEN’s effectiveness partly depends on excluding specific dietary components, a specialized diet, the Crohn’s Disease Exclusion Diet (CDED), was developed [31].

The CDED aims to provide an effective nutritional therapy for achieving disease remission, while allowing for a more pleasant and sustainable diet [30,31].

This diet reduces exposure to components of the Western diet that are associated with inflammation, causing changes in the microbiota and intestinal permeability. The direct intention of this exclusion diet in regard to dysbiosis is to help restore the intestinal barrier, promote an adequate immune response, extinguish the inflammatory process, and lead to the healing of the mucosa [18].

The CDED involves avoiding or reducing exposure to specific dietary components, such as animal fat; dairy products; gluten; red and processed meats; simple sugars; and various food additives, like emulsifiers, artificial sweeteners, sulfites, carrageenan, and taurine. On the other hand, CDED includes mandatory foods to provide fiber and specific starches as substrates for generating short-chain fatty acids. It also incorporates low-fat animal protein sources to enhance the composition of the microbiome and reduce intestinal permeability [31,32,33].

CDED is often used in combination with partial enteral nutrition (PEN) and is structured into three distinct phases:

✓ Phase 1 (week 0 to week 6): During this phase, there are mandatory, allowed, and disallowed foods. Enteral formula provides 50% of total energy requirements.
✓ Phase 2 (week 7 to week 12): The list of mandatory and disallowed foods remains unchanged, while the list of allowed foods becomes less restrictive. Enteral formula provides 25% of total energy requirements.
✓ Maintenance phase (after week 13): No mandatory foods are specified in this phase. The list of disallowed foods remains the same, and the list of allowed foods becomes more flexible. Enteral formula continues to provide 25% of total energy requirements. Additionally, this phase includes instructions for incorporating free meals on weekends, which may include some initially excluded dietary components [31,33].

The CDED is a relatively new approach to CD, and there is a need for clinicians to further understand its potential.

The primary objective of this study was to evaluate the effectiveness of this novel therapeutic approach in achieving remission for active disease in both children and adults, considering the latest available evidence. Furthermore, we aimed to explore potential secondary outcomes, including any changes in nutritional status and improvements in quality of life.

## 2. Materials and Methods

This systematic review adhered to the PRISMA (Preferred Reporting Items for Systematic Reviews and Meta-Analyses) 2020 guidelines designed for systematic reviews and meta-analyses of clinical intervention studies [34,35].

The systematic review was registered on the PROSPERO platform, the International Prospective Register of Systematic Reviews, with the registration number CRD42022335076 [36], available from https://www.crd.york.ac.uk/prospero/display_record.php?ID=CRD42022335076 (accessed on 21 June 2022).

We conducted searches in several databases, including PubMed/MEDLINE, Cochrane Library, Scopus, and Web of Science. The search strategy employed the following keywords:

- ((Gastrointestinal diseases[MeSH Terms]) OR (inflammatory bowel diseases[MeSH Terms]) OR (IBD[Title/Abstract]) OR (crohn disease[MeSH Terms]) OR (crohn[Title/Abstract])) AND ((crohn* disease exclusion diet[Title/Abstract]) OR (CDED[Title/Abstract]) OR (partial enteral nutrition[Title/Abstract]) OR (PEN[Title/Abstract])).

The articles identified through the search were imported into Rayyan—Intelligent Systematic Review tool software [37].

Duplicate studies were identified and removed. The selection of studies for inclusion was conducted by evaluating the established inclusion and exclusion criteria, along with a review of their titles and abstracts by two independent reviewers. Any discrepancies in article selection were resolved through consensus, and, if necessary, a third reviewer provided a resolution for any remaining disagreements.

### 2.1. Inclusion and Exclusion Criteria

#### 2.1.1. Inclusion Criteria

- Date of publication: Studies published between 30 June 2014 and 2 August 2022.
- Population: Human participants, including children and adults with active CD.
- Outcome: CDED as a nutritional intervention for achieving remission in CD.
- Study Design: Case–control studies, cohort studies, clinical trials, and randomized clinical trials.
- Language: Studies published in Portuguese, English, or Spanish.

#### 2.1.2. Exclusion Criteria

- Studies that addressed nutritional therapy for CD in children and/or adults with active disease but did not involve the use of CDED.
- Studies that did not provide results or prevalence data.

To evaluate the risk of bias in the studies included in our analysis, we employed the Cochrane RevMan tool, specifically designed for the development of systematic reviews and meta-analyses [38].

For this assessment, we categorized the risk of bias as either high risk, low risk, uncertain risk, or not applicable. Two independent reviewers conducted a thorough and independent evaluation of all the included studies. They assessed the risk of bias in various domains, including selection, performance, detection, attrition, and reporting bias, to gauge the quality of the obtained evidence.

#### 2.1.3. Dietary Intervention

Although the term “CDED” already implies the incorporation of partial enteral nutrition, several papers documented patients undergoing CDED without PEN. As a result, researchers often use terms such as “CDED + PEN” to denote the application of both the diet and PEN, and “CDED exclusive” or “CDED alone” to refer to cases where patients follow the dietary protocol without PEN supplementation. This distinction aids in the clarification of the specific dietary regimens under investigation and allows for a more precise analysis of their respective effects on disease management and outcomes.

## 3. Results

### 3.1. Included Studies

The search across the mentioned databases resulted in a total of 300 findings.

A total of ten studies were considered eligible for inclusion. There was one study for which the two primary reviewers disagreed. This study was referred to a third reviewer, who, after a comprehensive evaluation, opted to exclude it due to the absence of data on disease remission.

**Figure 1:**
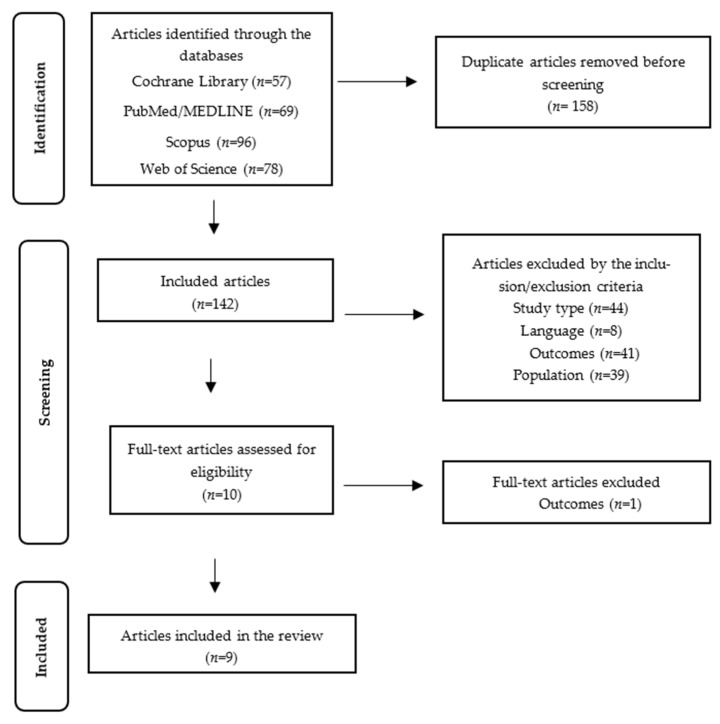
PRISMA flow diagram.

Consequently, nine studies were included in this systematic review. The study selection process is illustrated in Figure 1.

The included studies underwent a comprehensive analysis, collecting data on study type; participant number and age; implemented diet therapy; intervention duration; enteral nutrition formula utilized; and primary outcomes investigated, particularly clinical remission and response.

### 3.2. General Characteristics of the Studies

Three randomized clinical trials, three uncontrolled clinical trials, two retrospective cohort studies, and one prospective cohort study were included.

The total number of participants across these studies was 352.

The distribution of studies based on the age group of participants was five studies that focused exclusively on pediatric patients, two studies that included both children and adults, and two studies that included only adult patients.

It is worth noting that the study by Sigall-Boneh, in 2021 [39], used a sample from the clinical trial conducted by Levine, in 2019 [33], and this was accounted for in the presentation of the results.

### 3.3. Diet

Three studies compared the CDED in combination with PEN to EEN [39,40,41], while the remaining six studies implemented the CDED with PEN alone [31,32,33,42,43,44].

Among the studies using the CDED with PEN, three of them exclusively implemented the CDED alone in a smaller sample [31,32,42].

Urlep’s study, in 2019 [40], adapted the CDED, referring to it as the “anti-inflammatory diet for CD”. This adapted diet incorporated an enteral nutrition formula that provided 75% of the energy requirements. The adapted diet maintained CDED principles but was more restrictive, allowing only regionally and sustainably produced fruits, vegetables, meat, and fish.

Two studies adapted the CDED + PEN by implementing one or two weeks of EEN before starting the first phase of the CDED [32,41].

### 3.4. Outcomes

The primary outcomes were assessing the impact of the CDED in combination with PEN on the response and clinical remission of patients with active CD.

Remission was often categorized based on specific indices. In children and young people, remission was assessed using the PCDAI score [11] and, in one recent study, an adapted version known as the wPCDAI [43]. For adults, HBI [10] and/or the CDAI [8] were primarily employed to evaluate remission. In a study that included both children and adults [32], the HBI [10] score was used in conjunction with the Physician’s Global Assessment (PGA), which was evaluated based on predefined criteria.

Clinical response was assessed through changes in PCDAI/CDAI [8,11] and HBI [10] values. Additionally, the impacts on biochemical parameters such as fecal calprotectin, C-reactive protein (CRP), and erythrocyte sedimentation rate (ESR), all of which serve as inflammatory markers, were examined.

Urlep’s study, in 2019 [40], considered endoscopic clinical remission, evaluating the healing of the intestinal mucosa using the Simple Endoscopic Score for Crohn’s Disease (SES-CD) [44], a validated tool for this purpose.

The specific parameters defined for assessing remission and clinical response in each of the included studies can be found in Table 1.

In addition to evaluating remission and clinical response, the included studies also investigated other outcomes to assess the impact of the CDED, like diet tolerance (palatability, adherence, and side effects), changes in biochemical parameters (fecal calprotectin, ESR, and CRP), impact on anthropometric profiles (weight, height, and Body Mass Index), changes in intestinal microbiota, and influence on quality of life (symptom management, daily functioning, and well-being).

These additional outcomes provided a comprehensive perspective on the broader effects of CDED beyond remission and clinical response. Table 2 shows the main and secondary outcomes in each article.

### 3.5. Disease’s Remission

In the first clinical trial to implement the CDED, conducted by Sigall-Boneh in 2014 [31], a remission rate of 70.6% after six weeks was achieved. After 12 weeks, 84% of the patients in remission in the CDED + PEN group with follow-up were still in remission. Notably, seven patients received only CDED without a polymeric formula, and of those, six (85.7%) also achieved remission at week 12.

In a subsequent study conducted by Sigall-Boneh in 2017 [32], the impact of the CDED + PEN was assessed among individuals who had lost response to biological treatment. After six weeks, this approach led to remission in 13 of the 21 participants, with a remission rate of 61.9%. Similar to the initial study, some patients were treated with CDED alone, and of these, three out of four (75%) achieved remission.

The authors investigated the potential of initiating treatment with one or two weeks of EEN before transitioning to the CDED + PEN. This group achieved a remission rate of 60% at week 6, with no significant differences observed for the other groups. Among the 17 patients who had previously failed to respond to combination therapy (using biological drugs and immunomodulators), nine (53%) achieved disease remission with the CDED + PEN approach.

In the study conducted by Levine in 2019 [33], corticosteroid-free clinical remission was compared between a group of children receiving the CDED + PEN and a group receiving EEN. At week six, the group implementing the CDED + PEN achieved a significantly higher clinical remission rate (75%) compared to the EEN group (59%).

By week 12, the values remained similar in the CDED + PEN group (76%) compared to the EEN group (45%). This study concluded that children following the new nutritional approach experienced lower levels of inflammation and, as a result, achieved clinical remission without the need for corticosteroid therapy. Notably, the decline observed in children’s remission in the EEN group may be attributed to the introduction of a free diet implemented from the seventh week, while the CDED + PEN group maintained the CDED and continued to exclude dietary components that directly affect the intestinal mucosa.

Urlep, in 2019 [40], compared remission rates between an anti-inflammatory diet adapted from the CDED + PEN and the gold-standard treatment, EEN. Remarkably, both groups achieved a remission rate of 81.8% at week 6.

Sigall-Boneh, in 2021 [39], hypothesized that identifying patients with a rapid clinical response (around week three), associated with good adherence to diet therapy, could predict those who would achieve clinical remission at week six. A retrospective analysis of Levine’s clinical trial [33] showed that, of the 61 participants who achieved a clinical response at week three, 75.4% achieved clinical remission by week six.

Szczubelek, in 2021 [42], implemented the CDED + PEN and observed that 76.7% of participants achieved remission after 6 weeks, and this remission rate increased to 82.1% after 12 weeks.

In the study by Yanai in 2022 [45], it was found that 25 out of 40 participants (63%) achieved clinical remission at week six. Among those who achieved remission at week 6, a significant proportion (88%) maintained remission at week 12. Even at week 24, remission was sustained by 80% of the individuals. Remarkably, there were no significant differences observed at any time point between the group receiving the CDED + PEN and the group receiving CDED alone.

In the study by Matuszczyk in 2022 [46], it was observed that, out of the initial sample of 29 patients with active disease, 16 (55.1%) achieved remission after implementing the CDED + PEN for 12 weeks.

In Niseteo’s 2022 study [41], a disease remission rate of 68.9% was achieved, with no significant difference between groups. Notably, the CDED + PEN group had a slightly higher remission rate (75%) compared to the EEN group (65.9%). This study was one of three clinical trials that explored the possibility of starting with one or two weeks of EEN before implementing CDED + PEN, and it found no additional benefit in disease remission.

**Figure 2:**
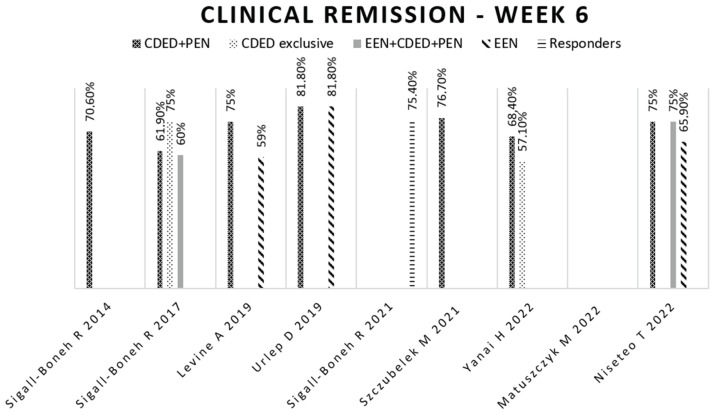
Clinical remission at week 6. Legend: CDED—Crohn’s Disease Exclusion Diet; EEN—exclusive enteral nutrition; PEN—partial enteral nutrition; Sigall-Boneh R, 2014 [31]; Sigall-Boneh R, 2017 [32]; Levine A, 2019 [33]; Urlep D, 2019 [40], Sigall-Boneh R, 2021 [39]; Szczubelek M, 2021 [42]; Yanai H, 2022 [45], Matuszczyk M, 2022 [46], Niseteo T, 2022 [41].

**Figure 3:**
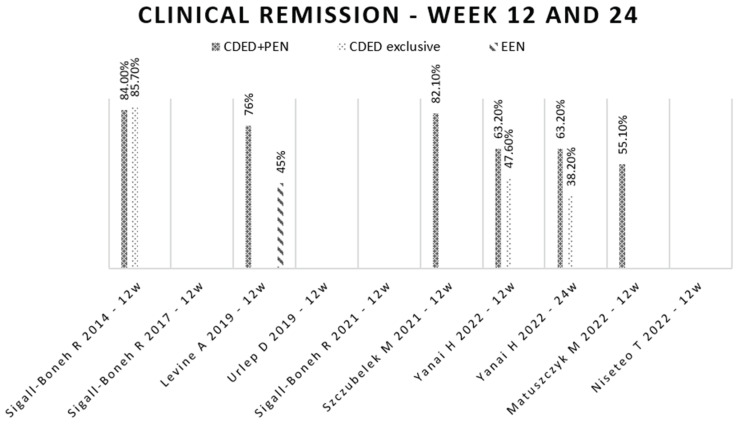
Clinical remission at weeks 12 and 24. Legend: CDED—Crohn’s Disease Exclusion Diet; EEN—exclusive enteral nutrition; PEN—partial enteral nutrition; Sigall-Boneh R, 2014 [31]; Sigall-Boneh R, 2017 [32]; Levine A, 2019 [33]; Urlep D, 2019 [40], Sigall-Boneh R, 2021 [39]; Szczubelek M, 2021 [42]; Yanai H, 2022 [45], Matuszczyk M, 2022 [46], Niseteo T, 2022 [41].

The results obtained for disease remission at weeks 6, 12, and 24 can be found in Figure 2 and Figure 3.

### 3.6. Clinical Response

Among all the studies included in the analysis, eight studies specifically evaluated clinical response as an outcome to be assessed [31,32,33,39,40,42,45,46].

Sigall-Boneh, in 2014 [31], observed a clinical response in 78.7% of patients after 6 weeks. Also, in the article by Sigall-Boneh in 2017 [32], a clinical response was observed in 90.4% of patients after 6 weeks.

Levine (2019) [33] verified a clinical response in 85% of patients in both groups (CDED + PEN vs. EEN).

Urlep, in 2019 [40], observed a clinical response in 91% of the EEN group and a response of 100% in the CDED + PEN group, with no significant differences between them.

In the study conducted by Sigall-Boneh in 2021 [39], through a retrospective analysis of Levine’s clinical trial in 2019 [33], it was found that 83.5% of patients achieved a clinical response at week three, with no significant difference between the CDED + PEN (82%) or EEN group (85%).

Szczubelek, in 2021 [42], achieved clinical response in 83% of patients after six weeks and 86% after 12 weeks.

Yanai, in 2022 [45], observed a clinical response in 74% of patients in the CDED + PEN group and in 67% of patients in the CDED-exclusive group, with no significant differences between them.

Matuszczyk, in 2022 [46], verified a clinical response in 69% of the patients after 12 weeks.

### 3.7. Tolerance

Only one of the included studies emphasized a major challenge to EEN therapy: the difficulty that many individuals face in maintaining this strategy for an extended period.

Levine’s study in 2019 [33] revealed that the CDED + PEN exhibited significantly higher tolerance, with 97.5% of users adhering to treatment. In contrast, EEN had a lower tolerance rate, i.e., of 73.7%.

### 3.8. Fecal Calprotectin

Levine’s study in 2019 [33] observed significant changes in fecal calprotectin levels. At week six, both the CDED + PEN group and EEN group showed a decrease in the mean calprotectin value compared to the baseline. There were no significant differences between the two groups at this point. However, between week 6 and week 12, the average calprotectin value continued to show a significant decrease in the CDED + PEN group (baseline: 3126 µg/g; week 6: 1744 µg/g; week 12: 732 µg/g), while the EEN group exhibited an increase in calprotectin levels (baseline: 2647 µg/g; week 6: 1021 µg/g; week 12: 1589 µg/g).

Urlep’s study in 2019 [40] also reported a significant decrease in the average value of fecal calprotectin in both the CDED + PEN and EEN groups, but the difference was non- statistically significant between them. In the CDED + PEN group, the fecal calprotectin mean values decrease from week 0 (426.5 µg/g) to week 6 (138.2 µg/g). In contrast, the EEN group had a less pronounced reduction (from week 0 (381.1 µg/g) to week 6 (206.9 µg/g)).

Szczubelek, in 2021 [42], reported a significant decrease in the level of fecal calprotectin during the intervention.

Yanai’s study in 2022 [45] observed a significant decrease in the fecal calprotectin value from 262 µg/g at baseline to 97 µg/g at week 12. There were no significant differences between the CDED + PEN and CDED exclusive groups. Importantly, 16 participants (40%) in the study achieved fecal calprotectin values below 100 µg/g.

In Matuszczyk’s study in 2022 [46], the reduction in fecal calprotectin level between week 0 and week 12 was significant. Among the children implementing the CDED + PEN, 35.4% of participants achieved normalization of fecal calprotectin levels (<250.00 µg/g) after 12 weeks. Additionally, 54.2% of the participants experienced a significant decrease of at least half in their calprotectin levels.

The results obtained for fecal calprotectin can be observed in Table 3.

### 3.9. Biochemical Parameters

In addition to fecal calprotectin, other biochemical parameters frequently assessed in these studies included CRP, ESR, and albumin. Remarkably, all the studies reported improvements in these parameters.

#### 3.9.1. C-Reactive Protein

Sigall-Boneh, in 2014 [31], reported a significant improvement in CRP levels compared with the baseline. After adopting the CDED, 21 out of 30 patients (70%) who were in remission and had previously elevated CRP values achieved normalization of their CRP levels (<0.5 mg/L).

Sigall-Boneh, in 2017 [32], also defined normal values for CRP <0.5 mg/L and observed a significant decrease in CRP values between baseline and weeks 6 and 12. No results were presented by the intervention group.

In Levine’s 2019 [33] study, the CDED + PEN group observed a significantly improved CRP value, from 23.6 mg/L at baseline to a mean value of 5 mg/L at week six. In the EEN group, the mean CRP value decreased from 24 mg/L at baseline to 4.1 mg/L at week six. At week 12, normalization of CRP (<5 mg/L) was observed in 75.9% of patients in remission in the CDED + PEN group, and in 47.6% of patients in the EEN group. There was no significant difference between the CDED + PEN and EEN in achieving normal CRP.

Urlep, in 2019 [40], also noted a decrease in the mean CRP values in both groups, without significant differences. The EEN group had an average CRP value of 16.5 mg/L at baseline, which decreased to 8.8 mg/L at week six. In contrast, the CDED + PEN group had an average CRP value of 18.4 mg/L at baseline, which decreased to 7.9 mg/L at week six. Notably, the author considered a negative CRP value of <8 mg/L, which was only found in the CDED + PEN group.

Szczubelek, in 2021 [42], also observed a significant reduction in the mean CRP values, which decreased from 4.30 mg/L at baseline to 1.30 mg/L at week six. At week 12, the average CRP value was 1.35 mg/L.

Yanai, in 2022 [45] reported that, among participants with elevated CRP levels (>5 mg/L), there was a significant decrease from baseline (14.5 mg/L) to week 6 (8.4 mg/L), and this improvement was sustained until week 24 (8.0 mg/L). Importantly, there were no differences in CRP levels between the CDED + PEN and CDED-exclusive groups at weeks 6, 12, or 24.

Matuszczyk, in 2022 [46], corroborated the findings of previous studies, highlighting the ability of the CDED + PEN to normalize CRP levels (<0.5 mg/L). The mean CRP value for the entire sample had a significant decrease from 1.0 mg/L at baseline to 0.20 mg/L at week 6 and remained low at 0.19 mg/L at week 12.

Niseteo, in 2022 [41], did not observe significant changes in the CRP value.

The results obtained for CRP can be observed in Table 3.

#### 3.9.2. Erythrocyte Sedimentation Rate

Sigall-Boneh, in 2014 [31], reported a significant improvement in the mean ESR values, from the initial value of 29.3 mm/h to 17 mm/h at week six.

Urlep, in 2019 [40], also observed a significant decrease in ESR values from baseline (EEN group—37.1 mm/h; CDED + PEN group—38.5 mm/h) to week six (EEN group—16.3 mm/h; CDED + PEN group—13.8 mm/h), with no significant differences between the groups.

Szczubelek, in 2021 [42], reported a non-significant decrease in the mean ESR value from baseline (9 mm/h) to week 12 (6 mm/h).

In Matuszczyk’s study in 2022 [46], the initial average ESR value was 21 mm/h, which had a significant decrease to 10 mm/h at week 6 and remained low (11 mm/h) at week 12.

The results obtained for ESR can be observed in Table 3.

#### 3.9.3. Albumin

Sigall-Boneh, in 2014 [31], noted a non-significant decrease in the initial mean albumin value at week 6 but a significant increase at week 12. The 36 participants who did not initiate immunomodulators and continued with diet therapy and follow-up improved the mean albumin value from 3.80 g/L at baseline to 4.12 g/L at week 12.

In a different clinical trial conducted by the same author in 2017 [32], a non-significant increase in the mean albumin value was observed from 3.5 g/L to 3.8 g/L in six weeks.

Urlep, in 2019 [40], found that both the EEN group and the CDED + PEN group experienced a significant increase in mean albumin levels, with no differences between the two groups. The EEN group showed an increase from baseline to week six, in the mean value of 3.2 g/L, while the CDED + PEN group had an increase of 3.5 g/L.

Szczubelek (2021) [42] and Yanai (2022) [45] reported that they did not observe any significant changes in albumin levels.

The results obtained for albumin can be observed in Table 3.

**Table 3:** Overview of the findings of fecal calprotectin, CRP, ESR, and albumin.

Study	Dietary Intervention	Fecal Calprotectin	CRP	ESR	Albumin
Sigall-Boneh, 2014 [31]	CDED + PEN CDED exclusive	NA	Significant decrease. No results available by intervention group.	Significant decrease. No results available by intervention group.	Significant increase. No results available by intervention group.
Sigall-Boneh, 2017 [32]	CDED + PEN CDED exclusive EEN + CDED + PEN	NA	Significant decrease in the total sample. No results available by intervention group.	NA	Non-significant increase. No results available by intervention group.
Levine, 2019 [33]	CDED + PEN EEN + free diet	Significant decrease. Non-significant differences between groups.	Significant decrease. Non-significant differences between groups.	NA	NA
Urlep, 2019 [40]	CDED adapted + PEN EEN	Significant decrease. Non-significant differences between groups.	Significant decrease. Non-significant differences between groups.	Significant decrease. Non-significant differences between groups.	Significant increase. Non-significant differences between groups.
Sigall-Boneh, 2021 [39]	CDED + PEN EEN	NA	Significant decrease. Non-significant differences between groups.	NA	
Szczubelek, 2021 [42]	CDED + PEN	Significant decrease.	Significant decrease.	Non-significant decrease.	No alterations were observed.
Yanai, 2022 [45]	CDED + PEN CDED exclusive	Significant decrease. Non-significant differences between groups.	Significant decrease. Non-significant differences between groups.	NA	No alterations were observed.
Matuszcz, 2022 [46]	CDED + PEN	Significant decrease.	Significant decrease.	Significant decrease.	NA
Niseteo, 2022 [41]	CDED + PEN EEN + CDED + PEN EEN	NA	Non-significant decrease in any group.	NA	NA

Legend: CDED—Crohn’s Disease Exclusion Diet; EEN—exclusive enteral nutrition; NA—not available; PEN—partial enteral nutrition.

### 3.10. Anthropometric Measurements

Most of the studies that evaluated the effect of diet therapy on weight and Body Mass Index (BMI) observed an increase in mean values, and there were no significant differences between the CDED + PEN and EEN groups [31,33,40,41,42,45,46].

However, Niseteo, in 2022 [41], found a significantly higher weight gain and increases in BMI in the CDED + PEN group compared to the EEN group.

### 3.11. Endoscopic Remission

In the study by Urlep (2019) [40], endoscopic remission was defined by an SES-CD [44] value ≤ 2. This study found that five (45.5%) of the patients, with no difference between the intervention groups, achieved endoscopic remission. Additionally, total mucosal healing, defined by an SES-CD [44] value of zero, was also assessed with no significant difference between groups. The EEN group had 45.5% of patients with total mucosal healing, while the CDED + PEN group had 27.3%.

In Yanai’s study in 2022 [45], endoscopic remission was defined as an SES-CD value of ≤3, which indicated complete healing of the mucosa. This study found that 42.1% of the patients in the CDED + PEN group and 28.6% of the patients in the CDED exclusive group achieved endoscopic remission (total mucosal healing) based on these criteria. No significant difference was noted in the proportion of patients who achieved endoscopic remission between groups.

### 3.12. Intestinal Permeability and Microbiota

Levine, in 2019 [33], assessed intestinal permeability in patients using a lactulose/mannitol (L/M) ratio test. This test involves ingesting a sweetened solution containing 5 g of lactulose and 1 g of mannitol and then analyzing urine to determine the level of intestinal permeability. The mean L/M ratio improved from 0.061 at week 0 to 0.012 at week three with the CDED + PEN but showed no significant change with EEN. At this point, 18 (69%) patients in the CDED + PEN group and 15 (56%) in the EEN group had normal intestinal permeability.

This study also examined changes in the microbiota after different dietary interventions. Stool samples from 70 patients were collected and analyzed, with 38 in the CDED + PEN group and 32 in the EEN group. The results showed a decrease in Proteobacteria in the EEN group, but this was reversed as soon as a free diet was introduced. In contrast, the CDED + PEN group did not experience this reversal; there was a decrease in Proteobacteria and an increase in Firmicutes.

### 3.13. Quality of Life

Szczubelek (2021) [42] assessed the effect of CDED + PEN on the quality of life of patients with CD. The study used the Inflammatory Bowel Disease Questionnaire (IBDQ) to measure the patients’ quality of life [47].

The results revealed a significant improvement in the quality of life of these patients at both week 6 and week 12 of the dietary intervention.

### 3.14. Bias Risk Analysis

**Figure 4:**
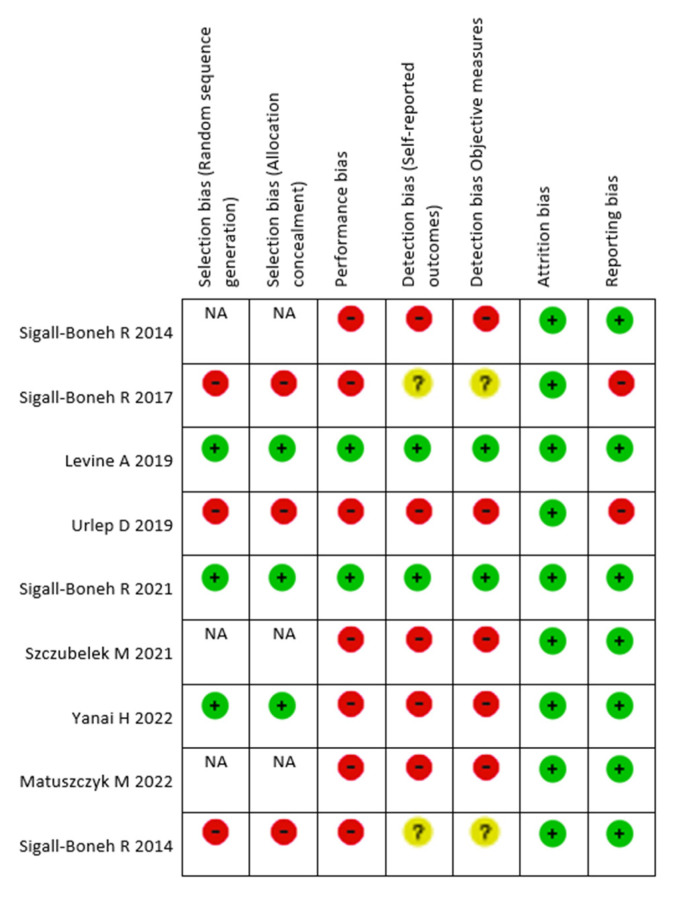
Risk of bias analysis for each included study. Legend: NA—Not Applicable; −—High Risk; +—Low Risk; ?—Uncertain risk; Sigall-Boneh R, 2014 [31]; Sigall-Boneh R, 2017 [32]; Levine A, 2019 [33]; Urlep D, 2019 [40], Sigall-Boneh R, 2021 [39]; Szczubelek M, 2021 [42]; Yanai H, 2022 [45], Matuszczyk M, 2022 [46], Niseteo T, 2022 [41].

To assess the risk of bias, Cochrane RevMan 5 software was used [38]. The results of the quality assessment of the clinical trials are shown in Figure 4.

Regarding selection bias, only the studies by Levine (2019) [33], Sigall-Boneh (2021) [39], and Yanai (2022) [45] were classified as low risk due to their randomized design and proper concealment of interventions during participant recruitment. Sigall-Boneh (2014) [31], Szczubelek (2021) [42], and Matuszczyk (2022) [46], were marked as “not applicable” for not using randomization. The rest of the studies had a high risk of selection bias. Sigall-Boneh (2017) [32] and Niseteo (2022) [41] did not employ randomization, assigning participants based on illness severity, and Urlep (2019) [40] allowed free choice in group selection.

Performance bias refers to knowledge of the allocated interventions by participants and personnel during the study. Most studies had a high risk, with both researchers and participants being aware of the diet therapy [31,32,40,41,42,45,46]. Only in the Levine (2019) [33] and Sigall-Boneh (2021) [39] studies did we observe a low risk of performance bias, as none of the health professionals involved in the research was informed about the specific diet therapy implemented for each individual. Additionally, assessments and contact were conducted by professionals external to the research team.

In the study by Niseteo in 2022 [41], there is no mention of whether the research team was aware of the allocated diet therapy for each group. However, we attribute a high risk of performance bias due to differences in methodology between the groups. Specifically, some participants did not adhere to the initial two weeks of EEN, and the second evaluation occurred at different times for each group.

Detection bias occurs when the outcome assessors are aware of the interventions assigned. This bias was prevalent in the majority of the studies. Levine (2019) [33] and Sigall-Boneh (2021) [39] were the only ones with low detection bias, as the researchers were blinded to the intervention. Sigall-Boneh (2017) [32] and Niseteo (2022) [41] lacked information for classification.

Attrition bias was low in all studies, with no incomplete data or potential result manipulation.

Reporting bias was generally low, as all specified outcomes were properly reported and analyzed, except in Sigall-Boneh (2017) [32] and Urlep (2019) [40], where adherence assessment relied on user feedback rather than validated methods.

No other biases were identified in the included studies.

## 4. Discussion

### 4.1. Disease’s Remission

According to the results of the included studies, this new therapeutic approach appears to be effective in inducing disease remission in both children and adults. However, it is crucial to determine whether it offers advantages compared to the standard treatment—EEN—especially in children [19]. Clinical trials comparing both approaches did not reveal significant differences.

When it comes to tolerance, Levine, in 2019 [33], found a significant difference in the CDED + PEN group compared to the EEN group.

In terms of achieving clinical remission, there was no significant difference between groups. The studies by Levine (2019) [33] and Niseteo (2022) [41] showed better results in the CDED + PEN group vs. EEN, but with non-significance rates.

The two retrospective cohort studies where EEN was applied before the CDED + PEN showed no significant difference in clinical remission rates [32,33,41]. This implies that the sequential utilization of these treatments may not confer any additional benefit. However, more studies with larger samples could be beneficial for future research into the impact of using the two therapeutic approaches at once.

An interesting finding emerged from clinical trials that exclusively used CDED, without PEN, as they achieved remission rates above 55% at week 6, 45% at week 12, and 35% at week 24 [31,32,45]. However, it is important to note that these studies had smaller sample sizes, and more research with larger samples and greater statistical power is needed to confirm these results.

Regarding the severity of the disease, it was observed that, in studies including patients with moderate-to-severe disease, Sigall-Boneh (2017) [32] and Szczubelek (2021) [42], clinical remission was achieved in 61.9% and 76.7% after six weeks, respectively. The limited number of participants in each study reduces the statistical power, and further research is required to determine whether disease severity can be a limiting factor in the effectiveness of this dietary therapy.

### 4.2. Biochemical Parameters, Intestinal Permeability, and Microbiota

Additionally, some more favorable results were identified in the CDED + PEN group regarding biochemical parameters.

The average value of fecal calprotectin was evaluated in five of the nine articles included, and all of them reported significant improvements [33,40,42,45,46]. Regarding the CRP value, this parameter was considered in all the studies included. With the exception of one article [41], all of them obtained significant improvements. ESR levels were assessed in four of the included studies [31,40,42,46], and only in one [42] was there non-significant improvement. In the articles where these biochemical parameters were assessed via the intervention group, there were no significant differences, suggesting that CDED is equally beneficial as an EEN for improving these parameters.

Regarding intestinal permeability and microbiota composition, Levine (2019) [33] observed more positive effects in the CDED + PEN group compared to the EEN group. The implementation of this diet therapy suggests a cause-and-effect relationship, indicating that the dietary components excluded in the CDED are potential agents that promote inflammation through intestinal dysbiosis. Excluding these components has a direct beneficial effect on the intestinal mucosa [15,17,33].

These findings underscore the role of diet in modulating the composition of the microbiota and its impact on the inflammatory processes, ultimately contributing to the management of CD and the achievement of mucosal healing, a critical goal in CD remission [17,48]. Interestingly, it was also noted that the mean fecal calprotectin value in Levine’s study in 2019 [33] increased in the group that transitioned from EEN to a free diet, emphasizing the negative effect that certain dietary components can have on the intestinal mucosa [15,18].

### 4.3. Duration of Intervention

The duration of the intervention and the need to maintain PEN are important factors to consider. Among the included studies, only one followed participant for longer than 12 weeks [45]. The results indicated potential benefits in providing 25% of energy needs with PEN during the maintenance phase of the CDED.

Notably, the clinical trial conducted by Sigall-Boneh in 2014 [31] followed 15 participants for a more extended period, from six months to two years. After the initial 12 weeks of CDED, the participants did not receive any specific diet therapy but were recommended to adhere to the principles of CDED, which aimed to reduce their exposure to dietary components that directly affect the microbiota. In this longer-term follow-up, it was observed that 73.3% of the participants who maintained their usual medication with immunomodulators did not require any adjustments, and 73.3% achieved complete healing of the mucosa.

To gain a more comprehensive understanding of the long-term effects of this dietary approach and whether it is necessary to continue PEN after 12 weeks, there is a need for more robust, randomized, investigator-blinded clinical trials with larger sample sizes and longer follow-up periods. Such studies can provide insights into the sustainability and effectiveness of this therapy over an extended timeframe.

### 4.4. Enteral Formula

The enteral nutrition formula most frequently used in the included studies was Modulen IBD by Nestlé Health Science. Modulen IBD is a complete polymeric powder formula enriched with TGF-β2, which plays a significant role in regulating the immune response and, consequently, the inflammatory processes [49]. However, it is important to note that the Modulen IBD formula contains some components that do not align with the principles of the CDED, such as milk fat, soy lecithin as an emulsifier, and sugars. It appears that the presence of these components in limited amounts did not have a significant impact on the results [18,28].

It is plausible that the beneficial effect of the dietary therapy is independent of the specific formula used, as suggested by the equally promising remission results in clinical trials that included a group with CDED exclusively [31,32,45].

Additionally, in the three studies that employed formulas other than Modulen IBD, no significant differences in remission and clinical response were reported [31,32,41].

This further underscores the need for more studies that directly compare the use of exclusive CDED with CDED + PEN to provide more robust evidence of the potential benefits of this therapeutic approach. Such comparative studies could help clarify whether the choice of formula is a critical factor or if the underlying dietary principles are the primary drivers of the positive outcomes observed.

### 4.5. Nutritional Status

It is essential to note that a comprehensive assessment of nutritional status was not thoroughly conducted in the included studies. Some studies only assessed weight [31,33,41] or BMI [40,42], and while there was a general improvement in these parameters, the results were not significant in any of the studies. Only one study evaluated biochemical parameters such as iron, vitamin, and calcium levels, and it found that the implementation of CDED + PEN optimized these values [42].

Additionally, in two studies, a significant improvement in the total albumin value was observed [31,40].

It is evident that there is a need for more specific study designs that extensively evaluate nutritional status during and after the implementation of this diet.

### 4.6. Importance of Registered Dietitians

It is worth emphasizing that all the authors of the included studies have stressed the importance of nutritional monitoring at various phases of the diet as a crucial factor in the success of the therapy. Support materials, such as explanations of the diet, lists of permitted and forbidden foods, recipes, and the availability of a registered dietitian’s team, have proven to be fundamental for ensuring adherence to and the maintenance of the CDED.

### 4.7. Highlights of Our Review

This systematic review was conducted using a rigorous methodology with well-defined inclusion and exclusion criteria. It covered multiple databases and included all published studies that implemented CDED in children and/or adults with active CD.

The review involved two reviewers who analyzed the articles for selection, reducing the risk of bias and ensuring the quality and relevance of the selected publications. In cases of uncertainty or conflicting opinions, a third reviewer was involved in the study selection process. The review adhered to PRISMA recommendations [34,35] and was supported by a protocol submitted to PROSPERO [36] and approved under the registration number CRD42022335076. The Cochrane RevMan 5 software [38] was used to analyze the risk of bias in the included studies, enabling a more comprehensive analysis.

### 4.8. What Is New?

The primary objective of this systematic review was to explain and summarize the existing evidence of this new dietary approach. It is noteworthy that, during this work, the European Society for Clinical Nutrition and Metabolism (ESPEN) released new guidelines on Clinical Nutrition in Inflammatory Bowel Disease [50]. The results of this review appear to align with the most recent ESPEN recommendations, which suggest that the CDED + PEN can be a suitable alternative to EEN in children and young people with mild-to-moderate CD to achieve clinical remission. Regarding adulthood, the recommendations indicate that CDED should be considered, with or without PEN [50].

Until this publication, there was only one systematic review published [51], which includes a smaller number of studies, all of which are also incorporated into our review.

This is such an actual theme that, since we performed a database search, some other nutritional interventions with this dietetic approach were published. Some of them extended the research intervention, with most of the results being consistent with our findings and highlighting the positive effects of this dietary intervention on endoscopic remission and mucosal healing [52].

Whether in adults [53] or children [52,54,55], the CDED has consistently shown high remission rates over time.

This approach also revealed significant improvements in eating behaviors [56].

## 5. Conclusions

In conclusion, this dietary therapy is an emerging option to be considered for the management of CD. All of the included studies show evidence of remission and also benefits in other clinical parameters, showing this as an alternative to other more invasive interventions.

However, more robust studies are necessary, especially to evaluate its efficacy in achieving long-term remission, and the potential risks of nutritional deficits. Further research will be vital in fully understanding the benefits and potential drawbacks of the CDED and the CDED + PEN in the management of CD in pediatric and adult patients.

## Figures and Tables

**Table 1:** Criteria defined to assess remission and/or clinical response.

Study	Criteria for Clinical Remission	Criteria for Clinical Response
Sigall-Boneh, 2014 [31]	PCDAI ≤ 7.5 (without height criteria at the age of diagnosis) or HBI ≤ 3 + CRP (0.5 mg/dL)	Decrease ≥ 12.5 points in PCDAI or decrease ≥2 points in HBI or improvement in biochemical parameters (Hg, ESR, albumin, and CRP)
Sigall-Boneh, 2017 [32]	PGA score + HBI < 5	Decrease ≥3 HBI points or remission
Levine, 2019 [33]	PCDAI ≤ 10 or PCDAI ≤ 7.5 (without height criteria at the age of diagnosis)	Decrease ≥12.5 points in PCDAI or remission
Urlep, 2019 [40]	PCDAI ≤ 10	Decrease ≥15 points in PCDAI or endoscopic response (50% reduction in SES-CD score) or changes in PCDAI and SES-CD or improvement in biochemical levels (ESR, CRP, Hg, albumin, thrombocytes, and calprotectin) or improvement in anthropometric measurements (weight and BMI)
Sigall-Boneh, 2021 [39]	PCDAI ≤ 10	Decrease ≥12.5 points in PCDAI or remission
Szczubelek, 2021 [42]	CDAI <150	Decrease ≥100 CDAI points or statistically significant decrease in CRP and/or leukocyte levels or Improvement in QoL or improvement in biochemical levels (total protein, albumin, Vit D, Vit B12, folic acid, Na, K, Ca, Fe, and ferritin) or improvement in BMI
Yanai, 2022 [45]	HBI < 5 at week 6	Decrease ≥3 HBI points
Matuszczyk, 2022 [46]	PCDAI ≤ 10	Decrease ≥12.5 points in PCDAI or remission
Niseteo, 2022 [41]	wPCDAI ≤ 12.5	NA

Legend: Ca—calcium; CDAI—Crohn’s Disease Activity Index; ESR—erythrocyte sedimentation rate; Fe—iron; HBI—Harvey–Bradshaw Index; Hg—hemoglobin; BMI—Body Mass Index; K—potassium; NA—not applicable; Na—sodium; PCDAI—Pediatric Crohn’s Disease Activity Index; CRP—C-reactive protein; PGA—Physician’s Global Assessment; QoL—quality of life; SES-CD—Simple Endoscopic Score for Crohn’s Disease; Vit—vitamin; wPCDAI—adapted version from the PCDAI.

**Table 2:** Overview of the studies regarding the duration of the intervention, diet therapy, nutritional formula used, and main and secondary outcomes.

Study	Time of Intervention	Diet Therapy	Nutritional Formula	Main Outcomes	Secondary Outcomes
Sigall-Boneh, 2014 [31]	12 weeks	Group 1 (*n* = 40) 0–6 weeks: CDED + PEN 50% 7–12 weeks: CDED + PEN 25% Group 2 (*n* = 7) 0–12 weeks: CDED exclusive	Modulen, Nestlé, Switzerland Pediasure, Abbott, The Netherlands	Clinical response Clinical remission	Weight Biochemical parameters
Sigall-Boneh, 2017 [32]	6 weeks	Group 1 (*n* = 12) 0–6 weeks: CDED + PEN 50% Group 2 (*n* = 4) 0–6 weeks: CDED exclusive Group 3 (*n* = 5) 0–2 weeks: EEN 3–6 weeks: CDED + PEN 50%	Modulen, Nestlé, Switzerland Pediasure, Abbott, The Netherlands	Clinical response Clinical remission	-
Levine, 2019 [33]	12 weeks	Group 1 (*n* = 40) 0–6 weeks: CDED + PEN 50% 7–12 weeks: CDED + PEN 25% Group 2 (*n* = 38) 0–6 weeks: EEN 7–12 weeks: PEN 25% + free diet	Modulen, Nestlé, Switzerland	Tolerance	Clinical response Clinical remission Intestinal permeability Poor adherence Microbiota composition Weight Biochemical parameters Fecal calprotectin
Urlep, 2019 [40]	6 weeks	Group 1 (*n* = 13) 0–6 weeks: EEN Group 2 (*n* = 12) 0–12 weeks: Diet adapted from CDED + PEN 75%	Alicalm, Nutricia, The Netherlands	Clinical remission	Clinical response Endoscopic remission Anthropometric parameters Biochemical parameters Fecal calprotectin
Sigall-Boneh, 2021 [39]	6 weeks	Group 1 (*n* = 39) 0–6 weeks: CDED + PEN 50% Group 2 (*n* = 34) 0–6 weeks: EEN	Modulen, Nestlé, Switzerland	Rapid clinical response	Clinical remission
Szczubelek, 2021 [42]	12 weeks	Total sample (*n* = 32) 0–6 weeks: CDED + PEN 50% 7–12 weeks: CDED + PEN 25%	Modulen, Nestlé, Switzerland	Clinical remission	Clinical response Anthropometric parameters Biochemical parameters Quality of life Fecal calprotectin
Yanai, 2022 [45]	24 weeks	Group 1 (*n* = 19) 0–6 weeks: CDED + PEN (1000 kcal) 7–12 weeks: CDED + PEN (600 kcal) 13–24 weeks: CDED + PEN (600 kcal) or CDED exclusive Group 2 (*n* = 21) CDED exclusive	Modulen, Nestlé, Switzerland	Clinical remission	Corticosteroid-free remission Clinical response Endoscopic remission Biochemical parameters Fecal calprotectin Anthropometric parameters Adherence
Matuszczyk, 2022 [46]	12 weeks	Total sample (*n* = 48) 0–6 weeks: CDED + PEN 50% 7–12 weeks: CDED + PEN 25%	Modulen, Nestlé, Switzerland	Normal fecal calprotectin after 12 weeks	Clinical response Clinical remission Biochemical parameters Anthropometric parameters
Niseteo, 2022 [41]	6–9 weeks	Group 1 (*n* = 16) 0–1/2 weeks: EEN 2/3–8/9 weeks: CDED + PEN 50% Group 2 (*n* = 4) 0–6 weeks: CDED + PEN 50% Group 3 (*n* = 41) 0–6/8 weeks: EEN	Modulen, Nestlé, Switzerland Pediasure, Abbott, The Netherlands NutriniDrink, Nutricia, The Netherlands Resource Jr, Nestlé, Switzerland Resource 2.0, Nestlé, Switzerland	Clinical remission	Anthropometric parameters

Legend: CDED—Crohn’s Disease Exclusion Diet; EEN—exclusive enteral nutrition; Jr—junior; PEN—partial enteral nutrition.

## Data Availability

The protocol for this systematic review was registered in the International Prospective Register of Systematic Reviews (PROSPERO) under the registration number CRD42022335076.
